# Red Blood Cell Aggregation, Angiogenesis and Hypoxia Biomarkers in Pancreatic Cancer

**DOI:** 10.3390/jcm15114109

**Published:** 2026-05-26

**Authors:** Maciej Wiewiora, Dorian Andreade, Christian Heiliger, Konrad Karcz

**Affiliations:** 1 Department of General Surgery, Vascular Surgery, Angiology and Phlebology, Faculty of Medical Sciences in Katowice, Medical University of Silesia, 40-055 Katowice, Poland; 2 Department of General, Visceral and Transplantation Surgery, Ludwig-Maximilians-University (LMU), 80539 Munich, Germany; dorian.andrade@med.uni-muenchen.de (D.A.); christian.heiliger@med.uni-muenchen.de (C.H.); drkarcz@me.com (K.K.)

**Keywords:** red blood cell, aggregation, angiogenesis, hypoxia, pancreatic cancer

## Abstract

**Background/Objectives:** This study aimed to investigate the effect of pancreatic ductal adenocarcinoma (PDAC) on the alterations in red blood cell aggregation related to angiogenesis and hypoxia markers. **Methods:** We studied 31 patients with confirmed PDAC. The aggregation of red blood cells (RBCs) was evaluated using a Laser-assisted Optical Rotational Cell Analyzer (LORCA). Serum vascular endothelial growth factor (VEGF) and hypoxia-inducible factor 1α (HIF-1α) levels were measured using ELISA. We estimated the following parameters specific to the aggregation process: the aggregation index (AI), the aggregation half-time (t_1/2_), and the threshold shear rate (γ_thr_). **Results:** All measured RBC aggregation parameters among PDAC subjects differed from those in the controls. The AI (*p* < 0.05) and γ_thr_ (*p* < 0.005) were significantly higher in the PDAC group, whereas t_1/2_ (*p* < 0.01) and AMP (*p* < 0.001) were significantly lower compared to the control group. The levels of VEGF (*p* < 0.0001) and HIF-1α (*p* < 0.0001) were significantly higher in the PDAC group than in the control group. There were significant correlations between RBC aggregation parameters and VEGF and HIF-1α. Multivariate analyses further identified t_1/2_ (*p* < 0.01) and γ_thr_ (*p* < 0.05) as independent predictors for VEGF. For HIF-1α, t_1/2_ (*p* < 0.05) was confirmed as an independent predictor. **Conclusions:** The results suggest, but do not demonstrate, a direct pathophysiological link between PDAC-associated hypoxia/angiogenesis and erythrocyte aggregation. Further studies are needed because the relationship linking PDAC to these aggregation indices is unclear.

## 1. Introduction

Pancreatic carcinoma (PC) is an aggressive malignancy characterized by a very poor five-year survival rate, representing one of the leading causes of cancer-related deaths in both the United States [1] and Europe [2]. The predominant histological subtype is PDAC, which accounts for approximately 85–90% of all pancreatic cancer cases. PDAC demonstrates particularly aggressive behavior, including pronounced local invasion, early distant metastasis, frequent recurrence, and limited eligibility for surgical resection [3]. Due to the absence of specific symptoms or the presence of only nonspecific clinical manifestations in the early stages, only 15–20% of patients are diagnosed at a point when potentially curative surgery is feasible [4].

Regardless of the underlying cause of malignancy, tumor development and progression are closely linked to extensive neovascularization, encompassing both microvessel formation and remodeling of the vascular network. VEGF serves as a central regulator of tumor angiogenesis and is upregulated by oncogene activation, various growth factors, and hypoxic conditions [5]. Hypoxia stimulates the release of signaling molecules and cytokines, while also increasing the expression of selected microRNAs involved in cancer progression [6]. These processes contribute to alterations in tumor suppressor gene expression, enhancement of angiogenesis, and modulation of immune escape mechanisms within the hypoxic tumor microenvironment. Recent investigations have demonstrated that PDAC is associated with elevated levels of VEGF and overexpression of HIF-1α in both blood and tumor tissue [7,8]. HIF-1α is recognized as a key regulator of the cellular response to hypoxia and plays a significant role in PDAC progression [9]. It has also been explored as a potential marker for prognostic stratification [10] and survival prediction in patients with PDAC [11]. Furthermore, several studies suggest a link between disturbances in RBC rheology and angiogenic activity, although the physiological relevance of this association remains insufficiently clarified [12,13].

Disturbances in RBC aggregation are known to be associated with various pathological conditions, including cardiovascular, metabolic, and hematological diseases, as well as infections [14]. Previous studies have also reported abnormalities in blood rheology in certain malignancies, including increased blood viscosity, enhanced RBC aggregation, and a reduced capacity for aggregate disassembly [15,16].

The aim of this study was to evaluate the influence of PDAC on changes in RBC aggregation in relation to biomarkers of angiogenesis and hypoxia.

## 2. Materials and Methods

### 2.1. Patient Characteristics

We studied patients with confirmed PDAC. To minimize the potential confounding effects on the rheological blood parameters, patients presenting with conditions that might influence these measurements were excluded, comprising diabetes mellitus, chronic kidney disease, anemia, coagulation abnormalities, uncontrolled arterial hypertension, a history of deep vein thrombosis of the lower limbs, and ongoing antithrombotic therapy. Patients with active inflammation, defined as a C-reactive protein (CRP) increase above the reference range, were also excluded from the study. The control group was composed of healthy individuals without diabetes mellitus, arterial hypertension, or any of the aforementioned conditions. The study protocol received approval from the Ethics Committee of the Medical University of Silesia, and written informed consent was obtained from all participants prior to enrollment.

### 2.2. Hemorheological Measurements

Venous blood samples were obtained from the antecubital vein using a syringe for biochemical analysis and anticoagulated with K_3_EDTA (1 mg/mL) for hemorheological assessment. Collection was performed between 8:00 and 9:00 a.m. following a 12 h overnight fast, and after the participants had rested for 10 min in accordance with rheological research requirements. All rheological measurements were carried out within two hours of sampling at a constant temperature of 37 °C. The hematocrit (Hct) was measured with a microhematometer. The Hct was adjusted to a standard Hct of 45%. Given the paramount importance of Hct on RBC aggregation, all samples were adjusted to 45% Hct to ensure that the aggregation results were not confounded by the baseline differences in Hct between groups. 

RBC aggregation and the aggregation kinetics were evaluated using a LORCA (Mechatronics, Zwaag, The Netherlands).

For the erythrocyte aggregation measurements, a laser backscatter light was used, in which the intensity of the reflection depended on whether the erythrocytes formed rouleaux at rest or if they were disaggregated and deformed at high shear rates. A blood sample was initially subjected to a high shear rate to cause cell disaggregation. Next, the motion was stopped to cause relaxation of the elongated RBCs and the forming of rouleaux. The LORCA computer program was used to analyze the aggregation parameters of RBCs, which were based on the syllectogram (i.e., the curve of the relation between the intensity of laser backscattering and time), where the light intensity is expressed in arbitrary units (au). The relevant part of the syllectogram reflects both static and kinetic parameters of aggregation and the behavior of RBC elongation. A normal syllectogram consists of an ascending part and a descending part, representing the progression in rouleaux formation. A decrease in laser backscatter intensity in the descending part of the syllectogram represents the progression of the aggregation process. The size of the ascending part of the syllectogram represents the time of relaxation of the elongated RBCs. The syllectogram amplitude depends on the size of both parts of the syllectogram; thus, it contains information about the aggregation process and the behavior of the elongated RBCs, which reflects the state where the elongated RBCs have recovered their normal shape.

The following aggregation-related parameters were derived from the syllectogram: AI in %; AMP in au; t_1/2_ in s, which characterizes the aggregation kinetics; and γ_thr_ in s^−1^.

### 2.3. Biomarker Measurements

Venous blood samples were obtained from the basilic vein in the morning (8:00–9:00 a.m.) after an overnight fast for biochemical analyses. The blood was collected into Vacutainer tubes. A total of 10 mL of whole blood was drawn into dry tubes to allow clot formation. Each specimen was promptly centrifuged at 3000× *g* for 30 min at 4 °C. Following the separation of cellular components, the serum was aliquoted and stored at −85 °C until further analysis. The study and control samples were processed concurrently. Serum concentrations of VEGF and HIF-1α were determined using an enzyme-linked immunosorbent assay (ELISA) with commercially available kits (VEGF R&D Systems ELISA, Minneapolis, MN, USA; HIF-1α ELISA Kit, MyBioSource, San Diego, CA, USA).

### 2.4. Biochemical Measurements

Red blood cell count and hematometric indices were determined using an automatic hematology analyzer (Pentra DX/DF 210, Horiba ABX Diagnostic, Grabels, France). Serum basic preoperative biochemical parameters and CRP levels were measured using an automatic analyzer COBAS INTEGRA 400 PLUS (Roche Diagnostic, Santa Clara, CA, USA). The level of carbohydrate antigen (CA 19-9) was measured using the automatic analyzer COBAS e411 (Roche Diagnostic, Santa Clara, CA, USA).

### 2.5. Statistical Analysis

Continuous data are expressed either as the mean ± standard deviation (SD) or as the median with interquartile range, when a normal distribution was not observed. Categorical variables are reported as counts and percentages. The normality of distribution for continuous variables was evaluated using the Shapiro–Wilk test. Between-group comparisons were conducted using the unpaired Student’s *t*-test or, in the case of non-normally distributed variables, the Mann–Whitney U test. Differences in categorical variables were assessed using the chi-square test or Fisher’s exact test when the expected cell counts were below five. Relationships between continuous variables were analyzed using Pearson’s test for normally distributed data or Spearman’s correlation coefficient for non-normally distributed data, as appropriate. The effect size for correlations was defined as weak (0.1 ≤ r_xy_ < 0.3), moderate (0.3 ≤ r_xy_ < 0.5), large (0.5 ≤ r_xy_ < 0.7) or very large (0.7 ≤ r_xy_ < 0.9). Independent predictors for angiogenesis/hypoxia parameters were determined via a multivariate regression model using the stepwise selection of rheological and biochemical parameters, with an entry criterion of *p* < 0.2. Potential predictors for multivariate modeling were initially identified via univariate.

A *p*-value below 0.05 was regarded as statistically significant. All statistical analyses were performed using the Statistica 12 software (StatSoft, Inc., Tulsa, OK, USA).

## 3. Results

A total of 31 patients with histologically confirmed PDAC, who were scheduled to undergo pancreaticoduodenectomy for a tumor located in the head of the pancreas, were included in this study. They consisted of 18 men and 13 women, with a mean age of 60.7 (SD 11.1) years. The control group was composed of 22 healthy individuals, 13 men and 9 women, with a mean age of 57.8 (SD 3.5) years. The baseline characteristics of the study population are presented in Table 1. There were no significant differences between the groups in the sex distribution, median body mass, or age; however, patients with PDAC tended to be older than the control group (*p* = 0.08). Significant differences were observed for several biochemical parameters between the two groups; in particular, glucose, platelet count, and bilirubin levels, as well as hepatic parameters and CA 19-9 antigen levels, were significantly higher in the PDAC group (Table 1). Total serum protein and albumin levels were lower in the PDAC group compared to the control group, but remained within the reference range. There were no significant differences in CRP and white blood cells (WBC) levels between the groups, nor were there differences in the proteinogram (including IgG levels). Red blood cell counts and hematological indices were similar between groups, with the exception of Hct. For the rheological measurements, all samples were adjusted to a 45% Hct to ensure that the aggregation results were not confounded by the baseline differences in Hct between the groups. No correlations were found between the biochemical parameters and aggregation parameters, including protein fractions.

The pathological characteristics of the patients are presented in Table 1. Jaundice occurred in 23 (74.2%) patients, and 6 (19.3%) patients underwent preoperative biliary drainage.

The levels of VEGF and HIF-1α were significantly higher in the PDAC group than in the control group (Table 2); the median VEGF level was four times higher, and the HIF-1α level was three and a half times higher in the PDAC group. All measured RBC aggregation parameters among PDAC subjects differed from those in the controls (Table 2). The AI was significantly higher in the PDAC, whereas the t_1/2_ and the AMP were lower compared with the control group.

**Figure 1:**
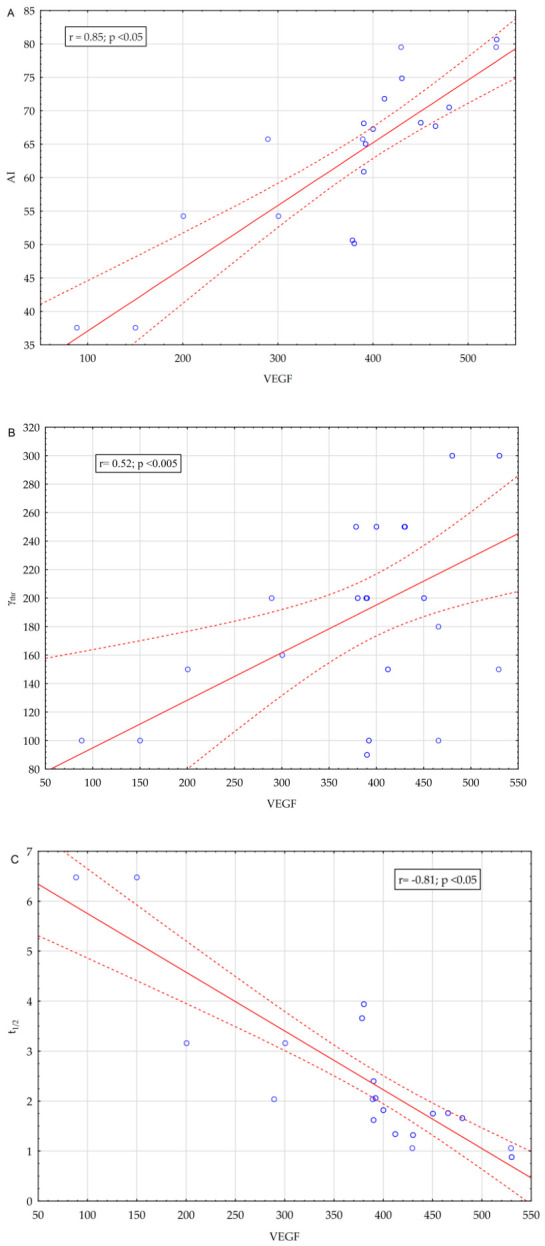
Correlation between VEGF and aggregation parameters: (**A**) with AI*; (**B**) with γ_thr_*; and (**C**) with t_1/2_*. * Spearman’s rank correlation.

**Figure 2:**
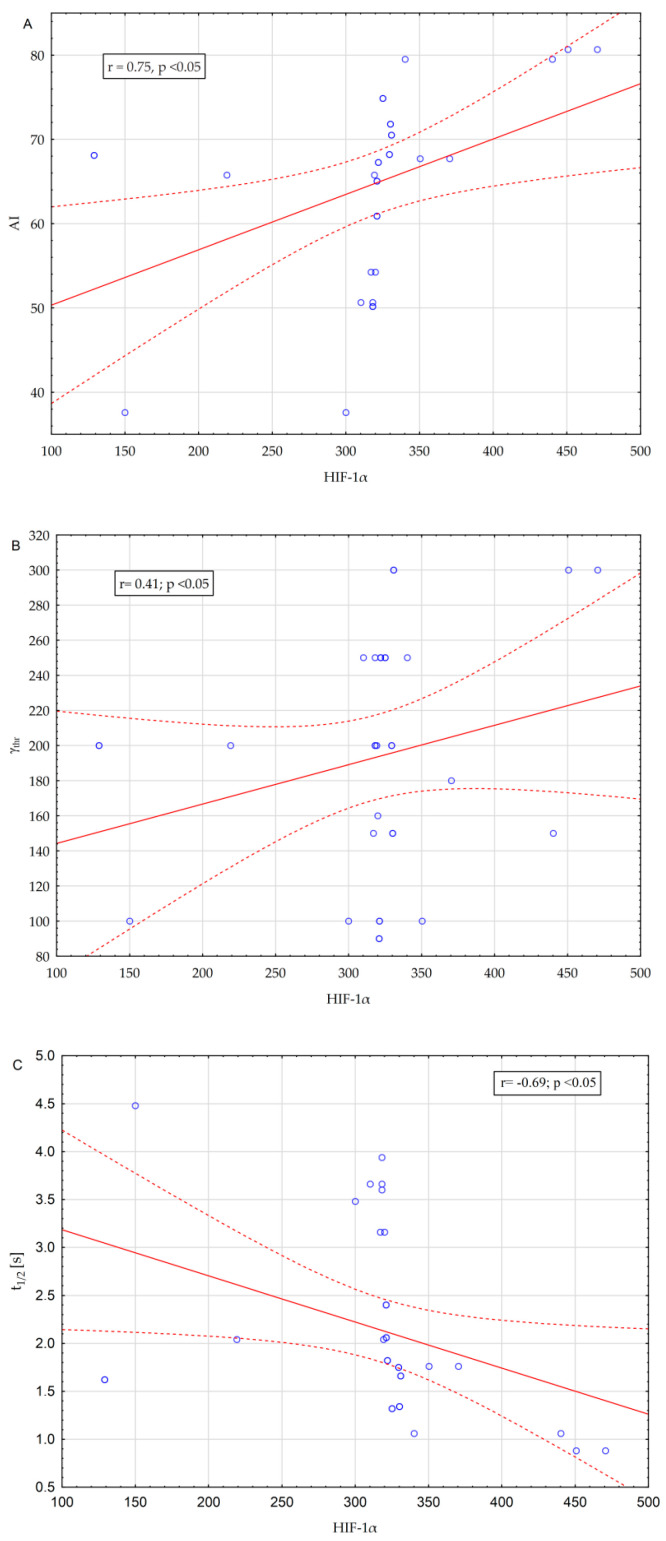
Correlation between HIF-1α and rheological parameters: (**A**) with AI*; (**B**) with γ_thr_*; and (**C**) with t_1/2_*. * Spearman’s rank correlation.

There were significant correlations between the RBC aggregation parameters and VEGF and HIF-1α. VEGF correlated positively with AI (r = 0.85, *p* < 0.05) and γ_thr_ (r = 0.52; *p* < 0.005) and negatively with t_1/2_ (r = −0.81; *p* < 0.05). HIF-1α correlated positively with AI (r = 0.75, *p* < 0.05) and γ_thr_ (r = 0.41; *p* < 0.05) and negatively with t_1/2_ (r = −0.69; *p* < 0.05). The effect size of the correlation was found to be moderate to large. The correlations between RBC aggregation and VEGF and HIF-1α are presented in Figure 1 and Figure 2. There was no correlation between RBC aggregation and the biochemical parameters.

Univariate analyses identified t_1/2_, AI, and γ_thr_ as potential predictors for VEGF, while t_1/2_, AI, and γ_thr_ were identified for HIF-1α. Multivariate analyses further identified t_1/2_ (β = −0.94; *p* < 0.01) and γ_thr_ (β = 0.21; *p* < 0.05) as independent predictors for VEGF. For HIF-1α, t_1/2_ (β = −1.33; *p* < 0.05) was confirmed as an independent predictor (Table 3).

## 4. Discussion

PDAC is considered one of the most aggressive solid malignancies, characterized by high mortality and very limited survival outcomes. A hallmark of PDAC is its profoundly immunosuppressive tumor microenvironment, which evolves throughout disease progression and is associated with the marked infiltration of myeloid-derived suppressor cells and tumor-associated macrophages [17,18,19,20]. Regardless of the underlying etiology, tumor expansion requires sufficient angiogenesis, primarily mediated by the upregulation of VEGF, because the tumor requires efficient blood perfusion for the supplementation of nutrition and oxygen in newly enlarged areas [21]. Moreover, increasing evidence indicates that HIF-1α serves as a central regulator of tumor hypoxia within the microenvironment and significantly contributes to PDAC progression [9,22]. HIF-1α has also been proposed as a potential marker for prognostic assessment [23] and survival prediction in patients with PDAC [11].

Adequate tissue perfusion, including within solid tumors, is governed by complex rheological determinants that influence microcirculatory blood flow. Numerous studies have demonstrated that RBC aggregation substantially affects blood viscosity under low shear conditions [24] and consequently modulates microvascular perfusion [25,26]. Effective microcirculatory flow is facilitated by the remarkable capacity of erythrocytes to deform in response to shear stress, as well as by the phenomenon of axial migration. Axial accumulation is caused by the axial migration of RBCs in the center of the flow stream, which results in the concentration of RBCs at the core and the development of a peripheral cell-free layer near the vessel wall that minimizes the resistance of blood flow [27]. The thickness of this cell-free layer increases with higher RBC aggregation and deformability, leading to a denser erythrocyte core and elevated local viscosity; nevertheless, the overall apparent viscosity may decline due to the lubricating properties of the expanded cell-free layer [28].

The results indicate that PDAC is associated with alterations in RBC rheology, expressed by an increased total aggregation extent and spontaneous ability to aggregate. Strict exclusion criteria were applied to construct a study group that allowed us to conclude that the results are a direct effect of PDAC, rather than a consequence of coexisting pathologies. The exclusion criteria accounted for factors that could significantly influence the rheological parameters under investigation. We believe that a cohort free of these confounding variables is more reliable and aligns closely with our primary objective: assessing the association between PDAC and the rheological parameters of red blood cells. Alterations in the kinetics of RBC aggregation expressed by shortening the aggregation half-time indicate that RBC aggregates form aggregates and rouleaux over a shorter time than in the control group. Thus, PDAC is associated with hyperaggregation of RBCs and increased spontaneous ability to aggregate compared to healthy individuals. The γ_thr_ value represents the minimal shear rate needed to prevent RBC aggregation and determines the ability of erythrocyte rouleaux, with an elevated γ_thr_ indicating higher aggregate stability in PDAC patients.

There was no correlation between RBC aggregation and the biochemical parameters, including protein fractions. RBC aggregation is determined by plasma protein composition and the surface properties of RBCs, with increased plasma concentrations of acute-phase reactants in inflammatory disorders being a common cause of increased RBC aggregation. Patients with active inflammation, defined as a CRP increase above the reference range, were excluded from the study. In addition, RBC aggregation tendency can be modified by alterations in RBC surface properties due to in vivo aging, oxygen-free radicals, or proteolytic enzymes.

PDAC patients exhibit a high ability to aggregate RBCs, which may contribute to problems with blood-flow conditions and result in regions of local hypoxia. This may decrease tissue blood perfusion and predispose one to tumor hypoxia, both known alterations in PDAC [29]. We found a significant correlation between RBC rheological parameters and both VEGF and HIF-1α. This relationship was supported by the multivariate regression results, which revealed that t_1/2_ and γ_thr_ are independent predictors for VEGF, whereas t_1/2_ is an independent predictor for HIF-1α. These results link rheological blood disturbances with angiogenesis and hypoxia markers in PDAC, but do not provide definitive proof; rather, they suggest a potential association.

There is well-established clinical evidence of impaired blood fluidity in individuals of advanced age, including increased plasma and whole-blood viscosity and enhanced RBC aggregation [30]. In our study, there were no significant differences in age between the groups; however, patients with PDAC tended to be older than those in the control group. We found no correlations between age and aggregation, angiogenesis, or hypoxia markers.

Previous studies demonstrated that RBC aggregation enhances their axial migration, as observed in ex vivo tube flow experiments [31] and in the microcirculation of animal models [32,33]. These findings describe a two-phase flow pattern characterized by a central core of aggregated erythrocytes surrounded by a cell-free plasma layer. The extent of this phenomenon is influenced by the strength of RBC aggregation within the microvascular network. The tendency of erythrocytes to aggregate, together with the presence of a slowly moving plasma layer, represents a key determinant of hydrodynamic resistance under low-flow conditions in both tubes [31] and capillaries [34]. The impact becomes more pronounced in cases of increased red blood cell hyperaggregation. Moreover, other researchers have suggested that RBC aggregation can modify the variability of the cell-free layer, particularly at low shear rates [35]. The influence of RBC aggregation on tissue perfusion is complex and multidirectional. Its hemodynamic effects may lead to either a reduction or an enhancement in tissue blood flow, depending on the interacting mechanisms [14], including axial RBC migration, changes in viscosity and resistance within the cell-free layer, alterations in microvascular hematocrit, and variations in wall shear stress. We found a significant correlation between RBC rheological parameters and both VEGF and HIF-1α. These correlations link rheological blood disturbances with markers of angiogenesis and hypoxia in PDAC. This relationship suggests that RBC hyperaggregation and the increased aggregate stability associated with PDAC may be critical factors driving flow disturbances within the tumor microcirculation, resulting in hypoxia and stimulating the overexpression of VEGF. It remains an open question whether changes in PDAC microcirculation and the accompanying hypoxia result solely from microenvironmental shifts that secondarily influence RBC aggregation. Changes in RBC aggregation may also stem from other comorbidities associated with PDAC and subsequently influence microcirculatory flow. This hypothesis is supported by our findings, which demonstrate that the overexpression of markers for angiogenesis and hypoxia is associated with RBC hyperaggregability. For the first time, we demonstrated that angiogenesis and hypoxia are linked to RBC rheological disturbances in pancreatic carcinoma. These hemorheological changes may cause impaired blood flow within the tumor’s microcirculatory network. Further studies are required to fully elucidate this relationship.

Recent research indicates that PDAC is linked to a range of interconnected biochemical, metabolic, and cytokine abnormalities [36]. However, due to the lack of comprehensive data addressing the association between PDAC and alterations in blood rheology, it remains challenging to identify which of these factors may play a leading role. Conversely, mechanisms such as hypoxia and activation of HIF-1 [37], as well as enhanced angiogenesis driven by upregulation of VEGF [21], are widely recognized contributors to multiple pathological conditions, including PDAC. Disturbances in blood rheological behavior have been reported in malignancies such as breast cancer, melanoma, and gynecological cancers [15]. The present study confirms hemorheological alterations, specifically enhanced RBC aggregation and evidence of a reduced capacity for disaggregation in PDAC. Furthermore, our results indicate existing relationships between angiogenesis and tissue hypoxia markers accompanying PDAC, based on systemic measurements of both angiogenesis markers and the hypoxia factor HIF-1. Naturally, these results cannot be unequivocally interpreted as a direct and unconditional relationship within the context of tumor tissue growth, and definitive confirmation will require further investigation.

### Study Limitations

This research was designed as an initial study to assess the effects of PDAC on the rheological properties of RBCs. Several limitations must be acknowledged. First, the results cannot be extrapolated to the general population due to the relatively small sample size. Furthermore, we examined the impact of PDAC on RBC rheology only at the time of diagnosis to explore potential relationships, but we did not assess patients post-surgery. The correlation results were related to angiogenesis and tissue hypoxia parameters measured in the blood. Comparing these parameters with those from tumor tissue after resection would have strongly emphasized their mutual interconnectedness. Finally, we did not examine other independent variables associated with PDAC that may influence RBC behaviors. Such an exploration would require a more homogeneous research model that incorporates surgical intervention.

## 5. Conclusions

In this study, we determined the hyperaggregation and increased spontaneous ability of RBCs to aggregate in PDAC patients. We also determined that circulating levels of angiogenesis and hypoxia biomarkers were significantly elevated compared to the control group. The results suggest, but do not demonstrate, a direct pathophysiological link between PDAC-associated hypoxia/angiogenesis and erythrocyte aggregation. Further studies are needed because the relationship linking PDAC to these aggregation indices remains unclear.

## Figures and Tables

**Table 1:** Baseline characteristics of the study group.

	PDAC (*n* = 31)	Control (*n* = 22)	*p*
Age (years)	60.7 (SD 11.1)	57.8 (SD 3.5)	0.08
Female	13 (41.9%)	9 (40.9%)	0.5
Male	18 (58.1%)	13 (59.1%)	0.4
Weight (kg)	65 (IQR 61–75)	73 (IQR 61–85)	0.19
RBC (10^6^/μL)	4.3 (SD 0.4)	4.5 (SD 0.8)	0.08
MCV (fl)	89.2 (SD 2.7)	91.1 (SD 3.3)	0.51
RDW (%)	12.9 (SD 2.9)	12.8 (SD 2.6)	0.78
Hb (g/dL)	12.3 (SD 1.4)	13.2 (SD 1.2)	0.02
Hct (%)	38.2 (SD 3.1)	41.1 (SD 2.5)	<0.001
WBC (10^3^/μL)	7.1 (SD 1.1)	6.5 (SD 2.9)	0.13
PLT (10^3^/μL)	315.1 (SD 74.2)	256.4 (SD 49.1)	0.003
CRP (mg/L)	3.1 (SD 0.8)	2.7 (SD 0.6)	0.76
Bilirubin, total (umol/L)	27 (IQR 9.3–77.3)	9.9 (IQR 6.6–13.2)	0.003
Glucose (mmol/L)	6.8 (IQR 5.4–7.3)	4.7 (IQR 4.2–5.1)	0.01
Creatinine (umol/L)	55 (IQR 46–70)	58 (IQR 51–70)	0.2
ALT (IU/L)	46 (IQR 20–105)	23 (IQR 11–29)	0.022
AST (IU/L)	36 (IQR 19–61)	18 (IQR 16–28)	0.022
Total blood protein (g/L)	66.8 (SD 5.6)	70.1 (SD 4.2)	0.008
Albumin (g/L)	39.8 (SD 2.1)	40.1 (SD 2.5)	0.02
α_1_-globulin (g/L)	2.3 (SD 02)	2.4 (SD 0.1)	0.37
α_2_-globulin (g/L)	9.2 (SD 0.7)	9.4 (SD 1.3)	0.41
β-globulin (g/L)	9.8 (SD 0.7)	9.6 (SD 0.5)	0.23
γ-globulin (g/L)	11.2 (SD 1.5)	11.1 (SD 1.5)	0.15
CA 19-9 (U/mL)	173 (IQR 5–606)	7 (IQR 2–30)	<0.0001
AJCC staging			
IA	3 (9.7%)		
IB	8 (25.8%)		
IIA	10 (32.2%)		
IIB	7 (22.6%)		
III	3 (9.7%)		

SD, standard deviation; IQR, interquartile range; RBC, red blood cell; Hb, hemoglobin; Hct, hematocrit; MCV, mean corpuscular volume; RDW, red cell distribution width; WBC, white blood cells; PLT, platelets; CRP, C-reactive protein; CA 19-9, carbohydrate antigen 19-9; ALT, alanine aminotransferase; AST, aspartate aminotransferase.

**Table 2:** Changes in RBC properties and blood biomarkers between the pancreatic cancer and control groups.

	PDAC (*n* = 31)	Control (*n* = 22)	*p*
AI (%)	64.1 (SD 10.3)	56.8 (SD 7.5)	0.03
AMP (au)	20 (IQR 18–23)	23 (IQR 20–25)	0.001
t_1/2_ (s)	1.8 (IQR 1.5–2.7)	3.2 (IQR 2–3.7)	0.0009
γ_thr_ (s^−1^)	200 (IQR 100–275)	100 (IQR 100–100)	0.003
VEGF (pg/mL)	397.5 (IQR 380–450)	92 (IQR 83–118)	<0.0001
HIF-1α (pg/mL)	321 (IQR 318–330)	90 (IQR 78–98)	<0.0001

SD, standard deviation; IQR, interquartile range; AI, aggregation index; AMP, amplitude; t_1/2_, aggregation half-time; γ_thr_, threshold shear rate; au, arbitrary units; VEGF, vascular endothelial growth factor; HIF-1α, hypoxia-induced factor 1α.

**Table 3:** Multivariate regression analysis of independent predictors of VEGF and HIF-1α.

	Parameter	β	SE_β_	*p*	R	R^2^	*p*
VEGF	t_1/2_	−0.94	0.31	<0.01	0.9	0.82	<0.0001
	γ_thr_	0.21	0.09	<0.05			
HIF-1α	t_1/2_	−1.33	0.57	<0.05	0.68	0.47	<0.001

β, beta coefficient; SE, standard error of the estimate; R, multiple correlation coefficient; R^2^, coefficient of multiple determination; HIF-1α, hypoxia induced factor 1α; VEGF, vascular endothelial growth factor; t_1/2_, aggregation half-time; γ_thr_, threshold shear rate.

## Data Availability

All data presented within this manuscript are available upon request from the corresponding author.
